# Functional Coping Dynamics and Experiential Avoidance in a Community Sample with No Self-Injury vs. Non-Suicidal Self-Injury Only vs. Those with Both Non-Suicidal Self-Injury and Suicidal Behaviour

**DOI:** 10.3390/ijerph14060575

**Published:** 2017-05-29

**Authors:** Emma Nielsen, Kapil Sayal, Ellen Townsend

**Affiliations:** 1 Self-Harm Research Group, School of Psychology, University of Nottingham, University Park, Nottingham NG7 2RD, UK; ellen.townsend@nottingham.ac.uk; 2 Division of Psychiatry and Applied Psychology, Institute of Mental Health, University of Nottingham, Innovation Park, Triumph Road, Nottingham NG7 2TU, UK; kapil.sayal@nottingham.ac.uk

**Keywords:** self-injury, self-harm, suicide, non-suicidal self-injury, experiential avoidance, coping

## Abstract

Although emotional avoidance may be a critical factor in the pathway from psychological distress to self-injury and/or suicidality, little is known about the relative importance of differing functional coping dynamics and experiential avoidance between people with self-injury histories of differing intent (e.g., Non-Suicidal Self-Injury only vs. Non-Suicidal Self-Injury plus Suicidal Behaviour; NSSI vs. NSSI + SB). A community-based survey (*N* = 313; female, 81%; ages 16–49 years, *M* = 19.78, *SD* = 3.48) explored self-reported experiential avoidance and functional coping dynamics in individuals with (i) no self-injury history (controls); (ii) a history of NSSI only; and (iii) a history of NSSI + SB. Jonckheere-Terpstra trend tests indicated that avoidance coping was higher in the NSSI and NSSI + SB groups than in controls. Emotion regulation was higher in controls than those with a history of self-injury (NSSI and NSSI + SB). Approach and reappraisal coping demonstrated significant ordered effects such that control participants were higher in these coping dynamics than those with a history of NSSI only, who, in turn, were higher than those with a history of NSSI + SB (Control > NSSI > NSSI + SB). Endorsement of the reappraisal/denial facet of experiential avoidance was most pronounced in those with a history of NSSI + SB (Control < NSSI < NSSI + SB). No significant ordered effects were observed for other dimensions of experiential avoidance. Understanding how the endorsement of functional coping dynamics and which components of experiential avoidance vary between groups with differing self-injury intent histories has important implications for treatment planning.

## 1. Introduction

Suicidal behaviours are strongly related to psychological distress [1,2]. Notions of avoidance and escape motives, related to this distress, are established in suicide science. Indeed, depiction of suicidality as an expression of avoidance is arguably congruent with concepts of suicide as escape from negative self-evaluation [3], conceptualization of “egression”—the ultimate escape [4] and the escape drive outlined in the Cry of Pain model [2].Research suggests that appraisals of an aversive internal experience as both (i) unbearable and (ii) needing to be extinguished are most relevant to suicidal behaviour, rather than the distress per se [1]. Indeed, some have argued the centrality of avoidance in explaining the nature of suicidality and the transition from distress to desperation [5]. Moreover, “unwillingness” or an inability to tolerate distress may be associated with suicidal urgency [5]. (The word “unwillingness” is commonly used in the literature pertaining to experiential avoidance. This is not to suggest that this is always something which is within an individual’s direct control.)

### 1.1. Experiential Avoidance

Experiential avoidance is defined as the tendency for non-acceptance or avoidance (e.g., escape, suppression, modification, control) of negative affective but not overtly dangerous states [6,7]. While consistent with established theoretical accounts, a specific association between experiential avoidance and suicidal intent has only recently begun to be developed [1].

Contemporary theoretical accounts of non-suicidal self-injury (NSSI) incorporate the role of experiential avoidance. For example, the Experiential Avoidance Model (EAM) [8] proposes that experiential avoidance plays a key role in explaining the cyclical re-engagement in NSSI. The EAM posits that a stimulus which elicits an intense aversive emotion may be conducive to a shift towards avoidance repertoires, such as engaging in self-injurious behaviours. The EAM suggests that NSSI is a coping strategy used to modulate unwanted or intolerable aversive mental states, or both. The resultant reduction in distress serves to maintain and strengthen the reliance on self-destructive behavioural repertoires; the avoidant behaviour is reinforced via temporary relief from, or elimination of, the internal state, and forms a self-perpetuating cycle. Thus, the avoidant behaviour becomes an automatic escape response.

While the EAM outlines specific predictions about NSSI, the conceptual account focuses exclusively on non-suicidal self-injury; “self-injurious behavior that occurs in the absence of any intent to die” [8] (p. 372), given evidence of “differences in the functions” of non-suicidal self-injury and suicidal behaviours (SB) (p. 373).

Recent research indicates that experiential avoidance may be an important psychological construct in self-injury, regardless of the suicidal intent associated with the behaviour [9]. However, to date, no research has explored whether individuals with differing intent histories (e.g., NSSI only, NSSI + SB) differ in endorsement of experiential avoidance. Exploring this is important, given indications that those who engage exclusively in non-suicidal self-injury may exhibit different psychological profiles to those engaged in self-injury of other intent [10].

Experiential avoidance can be considered as both a situation-specific behavioural ‘choice’ (internal or overt) and a dispositional tendency [11]. The current study assesses dispositional avoidance tendency across a number of broad domains: emotions, thoughts, memories, behaviours, autonomic sensations and pain. This multidimensional approach reflects research evidence suggesting that differing domains of experiential avoidance are differentially associated with psychopathology [11]. Therefore, multidimensional assessment has the potential to provide novel information regarding which aspects of avoidance are most commonly exhibited in, or are able to differentiate between, the three study groups. The measurement of dispositional, rather than situational, experiential avoidance is particularly suitable for this study given that we assessed for lifetime NSSI and suicidal behaviour. 

### 1.2. Functional Coping Dynamics

People with heightened levels of experiential avoidance tend to produce coping responses (e.g., cognitions, behaviours) that paradoxically lessen their ability to deal with stressful situations [12]. Given that (i) functionality has been outlined as differentiating non-suicidal self-injury from suicidal behaviour [8] and (ii) the endorsement of functional coping dynamics have been demonstrated to differentiate individuals with and without a lifetime history of self-injury [9], the current study also explores ordered effects in functional coping dynamics. That is to say, whether there are ordered effects in individuals’ beliefs about what the cognitions and behaviours they employ to cope with an identified stressor will allow them to do.

Functional accounts of coping are discernible from coping style accounts given their divergent emphases. While style accounts are primarily concerned with what the individual does, functional accounts are concerned with what these coping efforts give the individual, psychologically [13,14]. Cox and Ferguson [13,14] outline four functional coping dynamics: (i) approach coping; responses which permits an individual to deal directly with the stressor faced; (ii) avoidance coping; responses that the individual believes allows them to ignore the existence of the problem; (iii) emotional regulation coping; responses which an individual believes will allow them to deal with the emotional consequences of a stressor and (iv) reappraisal coping; responses which an individual believes allows them to readdress or reinterpret the meaning of the stressor faced.

There is an increasing emphasis on functionality as central in understanding self-injury [15,16]. However, at present, the literature addressing transactionally defined functional coping dynamics in relation to self-injury is extremely limited. Just two studies have assessed functional coping dynamics in relation to self-injury [9,16]. Participants with a lifetime history of self-injury, reported significantly higher levels of avoidance coping, as well as lower levels of approach, reappraisal, and emotional regulation coping, when compared with those who had never self-injured [9]. In individuals with a recent history of self-injury, functional coping dynamics were predictive of self-injury experience (or lack thereof), in response to a specific recent stressor. Reappraisal coping was predictive of self-injurious thoughts (ideation). Approach, emotion regulation, and reappraisal coping were predictive of self-injurious behaviour (enactment). Furthermore, emotion regulation coping differentiated self-injury ideation and enactment groups [16].

Both of the above studies [9,16] explore self-injury, regardless of suicidal intent. This ‘continuum conceptualisation’ encompasses NSSI, suicidal behaviour, and behaviour with mixed or ambivalent intent. This is consistent with recent taxometric analyses of the nature of suicidal intent [17]. However, whether functional coping dynamics vary between participants with differing intent histories remains unexplored. It is therefore important to understand how moderators of stress or psychological distress (e.g., coping) correspond to suicide intent history. 

### 1.3. Current Study

To further elucidate the role avoidance may play in suicidal behaviour amongst those with a history of self-injury, the current study investigates whether ordered effects are observed between those with no history of self-injury (“Control”), those with a history of non-suicidal self-injury only (“NSSI”) and those with a history of non-suicidal self-injury and suicidal behaviour (“NSSI + SB”). Inherent to the study is the premise that groups with no self-injury history (“Control”), non-suicidal self-injury only (“NSSI”), and non-suicidal self-injury plus suicidal behaviour (“NSSI + SB”) reflect a hierarchy or continuum of intent. We argue that this assumption is justified given that recent taxometric analyses of non-suicidal and suicidal self-injury demonstrate a continuum of intent [17].

As some have argued that suicidal behaviour is an “extreme form of emotional avoidance” [18], it is hypothesised that the ordered effects will be as follows: those with a history of suicidal behaviour plus non-suicidal self-injury will be higher in experiential avoidance than those with a history of non-suicidal self-injury only, who, in turn, will be higher in experiential avoidance than those with no history of self-injury (i.e., NSSI + SB > NSSI > Control).

Given the outlined centrality of aversive emotions, including (i) the appraisal of these experiences as intolerable and (ii) the drive to ameliorate these feelings and the situations that cause them, it is hypothesised that those with suicide behaviour plus non-suicidal self-injury (NSSI + SB) will be higher in avoidance and emotion regulation coping than those with a history of only non-suicidal self-injury (NSSI), who will, in turn, be higher in endorsement than those with no history of self-injury (avoidance, Control < NSSI < NSSI + SB; emotion regulation, control < NSSI < NSSI + SB). Conversely, it is hypothesised that those with no history of self-injury will be higher in reappraisal and approach coping than those with a history of NSSI only, who, in turn, will be higher than those with a history of suicide behaviour plus non-suicidal self-injury (reappraisal, Control > NSSI > NSSI + SB; approach, Control > NSSI > NSSI + SB).

## 2. Materials and Methods

### 2.1. Participants

A total of 318 adults completed the study. Five participants (1.6%) reported suicidal behaviour, but no history of NSSI (suicidal behaviour only). As there was insufficient statistical power to analyse participants reporting suicide attempt only, these five participants were removed from analysis. Therefore, a final sample size of 313 was achieved and all subsequent analyses reported pertain to this sample.

The majority of the sample were female (*n* = 252, 80.5%); 53 participants indicated that they were male (17.4%) and 8 participants indicated “other” or did not provide information (2.6%). Participants ranged in age from 16–49 years. However, the majority were young adults (*M* = 19.78, *SD* = 3.48). Five participants did not indicate their age (1.6%).

Of the 313 participants, 109 (34.8%; “Control”) participants reported no lifetime self-injury history. Over half of the sample had a history of non-suicidal self-injury only (53.0%; *n* = 166; “NSSI”), with a further 38 (12.1%; “NSSI + SB”) participants reporting a history of both non-suicidal self-injury and suicidal behaviour. 

### 2.2. Procedure

The questionnaires were administered in both online and paper format. The research project was advertised across a range of online platforms (e.g., Twitter, Reddit, Facebook etc.), the School of Psychology Research Participation Scheme (RPS) and poster advertisements across University campuses. University participants were recruited from a Russell Group University, UK. The majority of participants (87.5%) were recruited this way, via the University’s Research Participation Scheme. Participants were not screened for, nor excluded on the basis of, receiving clinical intervention(s), and no information regarding psychiatric diagnoses was obtained. Recruitment was not topic blind. Participants were not provided an inconvenience allowance. However, eligible participants (first year, undergraduate Psychology students at the host University) received RPS points. This represents partial course credit.

The study protocol was approved by the institutional ethics review board (reference 262R, date of approval 11 February 2013). All participants provided written informed consent (via computer). The protocol did not allow for parental/legal guardian consent to be obtained. Therefore, only participants aged 16 years or older were eligible to participate. All participants were provided with signposting information to sources of emotional support (e.g., Samaritans, UK).

### 2.3. Measures

#### 2.3.1. Demographic Factors

Age and gender demographics were collected.

#### 2.3.2. Self-Injury

Section one of the Inventory of Statements about Self-Injury (ISAS) [19,20] captures lifetime frequency of 12 non-suicidal self-injurious behaviours enacted “intentionally (i.e., on purpose) and without suicidal intent” (banging/hitting self, biting, burning, carving, cutting, wound picking, needle-sticking, pinching, hair pulling, rubbing skin against rough surfaces, severe scratching, swallowing chemicals). Additionally, the measure includes an “other” category, permitting free report of behaviour(s).

#### 2.3.3. Suicidality

Two items assessed engagement in suicidal behaviour. The first captured lifetime engagement (“Have you ever engaged in suicidal behaviours?”: yes, no), the second captured the recency of this behaviour “If you have selected YES to the above question, please indicate the last time you have done this: this week, this month, over one month ago but less than six months, six months or more). Single-item assessment capturing the presence or absence of suicidal behaviour are frequently used in suicide science [21]. Indeed, many commonly employed assessment tools (e.g., The Youth Risk Behavior Survey [YRBS]; The World Health Organization (WHO) Composite International Diagnostic Interview [CIDI]) contain single-item measures of suicide attempt, from which our question was adapted.

#### 2.3.4. Experiential Avoidance

The Multidimensional Experiential Avoidance Questionnaire (MEAQ) [11] assesses endorsement of experientially avoidant strategies, considering behaviours, thoughts and emotions. The 62-item measure captures experiential avoidance tendency across six dimensions: behavioural avoidance (overt, situational avoidance of physical discomfort and distress which prevents new learning e.g., “I go out of my way to avoid uncomfortable situations”), distress aversion (lack of openness towards internal experiences and non-acceptance of distress e.g., “The key to a good life is never feeling any pain”), procrastination (the delaying of anticipated distress e.g., “I try to put off unpleasant tasks for as long as possible”), distraction/suppression (the ignorance or suppression of distress e.g., “When something upsetting comes up, I try very hard to stop thinking about it”), repression/denial (lack of awareness about distress, distancing and dissociating from distress e.g., “I am able to ‘turn off’ my emotions when I don’t want to feel”), and distress endurance (willingness to persevere in the face of distress in the pursuit of values e.g., “I am willing to suffer for the things that matter to me”). Each item is assessed on a six-point Likert scale (“strongly disagree” to “strongly agree”). Dimensions load on a single factor.

Two items are reverse scored. Total score is calculated as the sum of scales, with distress endurance scale total reverse scored (behavioural avoidance + distress aversion + procrastination + distraction/suppression + repression/denial dimensions + [77 − distress endurance]). Higher total score indicates higher experiential avoidance. Internal consistency for all MEAQ subscales in the present study was good (behavioural avoidance, α = 0.873; distress aversion, α = 0.868; procrastination, α = 0.857; distraction/suppression, α = 0.863; repression/denial, α = 0.862; distress endurance, α = 0.855; overall, α = 0.925).

#### 2.3.5. Coping

The Functional Dimensions of Coping scale (FDC) [14] captures, both qualitatively and quantitatively, how individuals respond to difficult situations and what they believe their cognitions and behaviours will achieve. Sections one and two of the measure allow the participant to describe (i) the most stressful recent event they have experienced (in the previous three months) and (ii) the coping responses (i.e., cognitions, behaviours) employed to deal with this. Section three consists of a 16-item series of seven-point (“not at all” to “very much so”) Likert measures assessing the perceived function of these coping responses.

The measure captures four dimensions of coping: approach (dealing directly with the problem e.g., “To what extent did this/these activities allow you to understand something of the nature of the problem, from which you could behaviour to deal directly with it?”); avoidance (allowing the individual to ignore the existence of the situation e.g., “To what extent did this/these activities distract you from thinking about the problem?”); emotional regulation (dealing with the emotional consequences of a problem e.g., “To what extent did this/these activities allow you to manage the distress and upset caused by the event?”); and reappraisal functions (readdressing and reinterpreting the meaning of a situation e.g., “To what extent did this/these activities allow you to step back and look at the problem, in a different way, such that it seemed better?”).

Item scores are summed; higher scores indicate higher endorsement of coping function. Internal consistency for FDC subscales in the present study was good (approach, α = 0.862; avoidance, α = 0.828; emotion regulation, α = 0.805; reappraisal, α = 0.740).

### 2.4. Data Analysis

Data were analysed using SPSS v21 (SPSS Inc., Chicago, IL, USA) for Windows. All functional coping dynamics and distress aversion, procrastination, repression/denial and distress endurance subscales of the MEAQ were not normally distributed. Therefore, non-parametric tests were conducted. Cases were excluded pairwise throughout.

A series of Jonckheere–Terpstra trend tests were conducted to explore the predicted ordered effects within functional coping dynamics and experiential avoidance. The rank-based, non-parametric test assesses for the presence of pre-defined, ordered difference in medians in a stated direction. Thus, Jonckheere–Terpstra trend tests differ from alternative non-parametric approaches as they test a priori hypotheses regarding the relative endorsement of a construct between groups (e.g., A > B > C or A < B < C), rather than merely testing whether statistically significant differences are observed (e.g., groups A, B, and C entered into a Kruskal–Wallis test) [22,23]. When there is a priori ordering, Jonckheere–Terpstra trend tests have more statistical power than alternative non-parametric analyses (e.g., Kruskal–Wallis test). Jonckheere–Terpstra tests have previously been successfully employed to assess ordered effects in suicide science [24].

## 3. Results

### 3.1. Preliminary Analysis

Of the 313 participants, 109 (34.8%; “Control”) participants reported no lifetime self-injury history. Over half of the sample had a history of non-suicidal self-injury only (53.0%; *n* = 166; “NSSI”). All participants who indicated positively to an ISAS assessed form of NSSI and have never engaged in suicidal behaviours were included in the “NSSI” group, regardless of the number of reported episodes of NSSI. All analyses presented in text utilise this classification criterion. We subsequently re-ran all analyses, including only those participants who had engaged in NSSI behaviours more than five times in the “NSSI” group. The pattern of results remains unchanged for functional coping dynamics. In experiential avoidance we see significant ordered effects in behavioural avoidance (Control < NSSI < NSSI + SB [*n* = 284], T_JT_ = 13,577.00, Z = 2.12, *p* = 0.034) and distress endurance (Control > NSSI > NSSI + SB [*n* = 282], T_JT_ = 13,344.50, Z = 2.06, *p* = 0.039), in addition to the significant ordered effect observed in the repression/denial facet presented in the body of the manuscript), with a further 38 (12.1%; “NSSI + SB”) participants reporting a history of both non-suicidal self-injury and suicidal behaviour. 

Of those who had ever self-injured, the majority had done so 50 times or less (1–5 times, *n* = 26, 12.7%; 6–50 times, *n* = 82, 40.2%). Twenty-three participants (11.3%) reported 51–100 episodes of self-injury, 50 participants (24.5%) reported engaging in non-suicidal self-injurious behaviours 101–500 times, and 23 individuals reported a lifetime history greater than 500 behaviours (11.3%). Considering the recency of non-suicidal self-injury, around one-third of participants reporting lifetime histories of non-suicidal self-injury (*n* = 59; 37.3%) had self-injured within the six months prior to study completion (this week, *n* = 24, 15.2%; this month, *n* = 13, 8.2%; 1–6 months, *n* = 22, 13.9%). Of those who reported having ever engaged in suicidal behaviours, around one-quarter of participants (*n* = 10; 23.7%) reported this having occurred within the last six months (this week, *n* = 1, 2.6%; this month, *n* = 1, 2.6%; 1–6 months, *n* = 8, 21.1%). Recency data was missing for one participant.

To test for between-group differences, method versatility (number of lifetime methods of self-injury), lifetime frequency, and recency of self-injury were entered into binary logistic regression. These factors did not predict group membership (NSSI; NSSI + SB, see Table 1).

### 3.2. Ordered Effects in Experiential Avoidance

In order to assess whether experiential avoidance exhibited significant ordered effects, a series of Jonckheere–Terpstra trend tests were conducted. The ordered effect in overall endorsement of experiential avoidance did not reach statistical significance, *p* = 0.058. However, examination of the measure subscales indicated a significant ordered effect in repression/denial (see Table 2).

### 3.3. Ordered Effects in Functional Coping Dynamics

**Figure 1:**
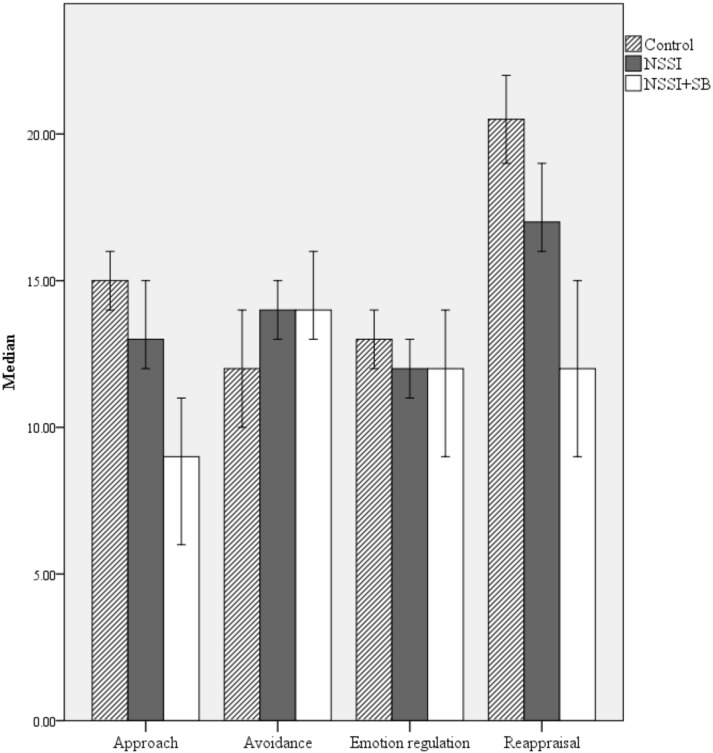
Median endorsement of functional coping dynamics across self-injury groups (Control vs. NSSI vs. NSSI + SB). Error bars = 95% CI.

As per the analyses conducted to explore the endorsement of experiential avoidance across groups, a series of Jonckheere–Terpstra trend tests were conducted in order to assess whether functional coping dynamics exhibited significant ordered effects. Results indicate statistically significant ordered effects in approach: Control > NSSI > NSSI + SB (*n* = 309), T_JT_ = 17,279.50, Z = 4.13, *p* < 0.001 (Median endorsements were 15.00 [*IQR* = 5.75], 13.00 [*IQR* = 8.00] and 9.00 [*IQR* = 9.00] respectively, see Figure 1), avoidance coping: Control < NSSI < NSSI + SB (*n* = 308), T_JT_ = 15,736.50, Z = 2.37, *p* = 0.018 (Median endorsements were 12.00 [*IQR* 8.00], 14.00 [*IQR*= 9.00] and 14.00 [*IQR* = 9.00] respectively), emotion regulation: Control > NSSI > NSSI + SB (*n* = 309): T_JT_ = 12,103.00, Z = −1.97, *p* = 0.048 (Median endorsements were 13.00 [*IQR* = 4.00], 12.00 [*IQR* = 6.00] and 12.00 [*IQR* = 8.00] respectively) and reappraisal coping: Control > NSSI > NSSI + SB (*n* = 309), T_JT_ = 17,223.50, Z = 4.01, *p* < 0.001 (Median endorsements were 20.50 [*IQR* = 6.75], 17.00 [*IQR* = 11.00] and 12.00 [*IQR =* 13.00]).

Here, it is important to note that the observed ordered effect in emotion regulation coping was in the opposite direction to that hypothesised. Further, while the ordered effects observed in avoidance and emotion regulation coping are statistically significant, the NSSI and NSSI + SB median remain tied. Consequently, the observed effect sizes are most compelling for approach (*r* = 0.23) and reappraisal (*r* = 0.23) coping.

## 4. Discussion

The current study is, to the best of our knowledge, the first to explore ordered effects in both experiential avoidance and functional coping dynamics between individuals with self-injury histories of differing intent (NSSI; NSSI + SB) and control participants with no history of self-injury. Results indicate that the endorsement of functional coping dynamics differs between self-injury groups. As self-injury group status (NSSI; NSSI + SB) was not predicted by versatility of methods, frequency, or recency of self-injury, it is unlikely that these factors can explain the above ordered effects, thus increasing our confidence that the observed differences relate to differences in intent history between groups.

As hypothesised, avoidance coping was higher in the NSSI and NSSI + SB groups than in controls. Converse ordered effects were observed in approach and reappraisal coping (Control > NSSI > NSSI + SB). Again, this is congruent with the hypothesised trends. However, contrary to our expectations, those with no history of self-injury (Control) were higher in emotion regulation coping than those with a history of NSSI or NSSI + SB; Control > NSSI > NSSI + SB. The directionality of trend observed should be considered in light of the measurement employed. While the Functional Dimensions of Coping scale (14) pertains to assess transactional functional coping accounts—what the person believed their coping responses to a given situation would achieve—the items contained within the emotion regulation scale (e.g., “To what extent did this/these activities allow you to manage the distress and upset caused by the event?”) arguably relate more readily to the efficacy of these coping efforts, rather than the underpinning motivational dynamics [9].

The observed effect sizes are more compelling for approach (*r* = 0.23) and reappraisal (*r* = 0.23) coping. These effect sizes are comparable to those reported elsewhere [9,16]. It is unsurprising that the observed effect sizes are modest, given that the FDC assesses transactionally defined coping in response to a single, recent event.

Considering dispositional experiential avoidance, contrary to our hypotheses, no significant ordered effect was observed for total MEAQ score. This could be seen to challenge the assertion that suicidal behaviour can be conceptualized as an “extreme form of emotional avoidance” [18] (p. 59). When considering the constituent facets of experiential avoidance assessed in the multidimensional measure, again, no significant ordered effects were observed in distress aversion, procrastination, distraction/suppression scales or distress endurance scales. However, significant ordered effects were observed in repression/denial (Control < NSSI < NSSI + SB). This indicates that those with a history of both non-suicidal self-injury and suicidal behaviour report higher levels of distancing and dissociating from distress when compared to those with a history of non-suicidal self-injury only and, in turn, those with no self-injury history. This is important for clinicians who carry out assessments following an episode of self-injury to be aware of. However, we must remain mindful that inferences of causality cannot be drawn.

The MEAQ considers responding to experiences such as autonomic sensations and pain. Alternative, and commonly employed, assessments of experiential avoidance do not necessarily pertain explicitly to these domains, focusing more on emotionality (e.g., Acceptance and Action Questionnaire II, AAQII) [25]. Our findings are suggestive of the potential importance of assessing dispositional experiential avoidance across a number of domains, given the differing patterns of significance observed across dimensions. 

Our results may have important implications for intervention planning. It is crucial that all those who self-injure and are in distress are met with compassionate and appropriate care, not least given the strong association of these behaviours with death by suicide (regardless of the suicidal intent of self-injury) [26]. Taken together, our results suggest that functional coping dynamics might be important factors to consider in future research to inform clinical practice. Further to this, our results indicate that exploring experiential avoidance—as an indiscriminate response—may have clinical utility. While several existing treatment programs for suicidal behaviour aim to reduce avoidance (e.g., Mindfulness-Based Cognitive Therapy; MBCT), our results suggest which facets of experiential avoidance may be principle targets for intervention planning. Considering the observed ordered effects in dispositional experiential avoidance, results indicate the importance of attending to aversive states and accepting negative experiences, particularly in the pursuit of goals and values.

### Limitations

The current study must be interpreted within the context of a number of limitations. The primary limitation of the work is the cross-sectional design, precluding the ability to make inferences regarding causality. Furthermore, the study relied on self-report methodology. This may be subject to recall biases, which may be particularly marked when reporting on items falling within the repression and denial facet of experiential avoidance, given that these dissociative tendencies are considered to be largely outside of an individual’s awareness [11]. Future research may seek to address these limitations by employing longitudinal designs and/or comparing the endorsement of self-reported items and performance on behavioural measures.

The majority of the participants within the current study were young and recruited via the University’s research participation scheme. In future research, it will be important to evaluate if these findings generalise to more diverse samples, especially given evidence that endorsement of experiential avoidance and functional coping is correlated with age [9]. Future work may also benefit from assessing psychiatric diagnoses; exploring whether the observed ordered effects remain consistent across individuals with different primary diagnoses would further self-injury science.

The current study compared those with a history of self-injury with and without a history of suicidal behaviour. It is acknowledged that some people who engage in suicidal behaviours may have no history of non-suicidal self-injury. To provide a comprehensive account of experiential avoidance and functional coping dynamics in relation to NSSI and suicidal behaviour, the exploration of this fourth group (SB only) would be of paramount importance. Future research could therefore extend the current study by sampling in such a way that the necessary statistical power is achieved to perform further, more nuanced, subgroup analyses. This could be achieved by a number of means, including utilising targeted sampling strategies (e.g., recruiting via support services/forums) and sampling from clinical services. This may also increase the sample size of the NSSI + SB group, which, in the current study, remains moderate. The addition of an SB only group, in conjunction with an increased NSSI + SB group size, may allow for clearer elucidation of ordered effects for avoidance and emotion regulation coping.

While the current study outlined recency and frequency to typify the sample, we did not explore the endorsement of experiential avoidance and coping functions between individuals with different recency of experiences. This research could therefore be extended to explore differing characteristics of self-injury (e.g., recency, frequency, method, etc.) in subgroup analyses. This more comprehensive analysis would provide valuable insight into the psychological and functional coping dynamics underpinning change in these complex behaviours.

The study employed a single-item assessment of suicidal behaviour (with an additional item probing recency). While recent epidemiological reviews indicate that the majority of research in the field employs single-item assessment [21], and our question was adapted from two commonly employed instruments (YRBS; CIDI), it is acknowledged that multiple item questioning may prove more comprehensive, revealing both false-positive and false-negative responding [27]. This is something to consider when planning future research.

## 5. Conclusions

Notwithstanding these limitations, the study offers a novel contribution to the literature addressing the role of experiential avoidance and coping in self-injury behaviour, for the first time exploring ordered effects comparing those with no self-injury history, those with a history of non-suicidal self-injury only, and those with a history of non-suicidal self-injury and suicidal behaviour. Taken together, these results suggest that functional coping dynamics (approach, avoidance, emotion regulation, reappraisal) differentiate those with differing self-injury histories (control vs. NSSI vs. NSSI + SB). Further, the study elucidates which component aspects of experiential avoidance may also be important; while no significant ordered effects were observed in total MEAQ score, a significant trend was observed in the repression/denial dimension of experiential avoidance. This has implications for future clinical research and treatment planning—specifically perhaps for exploring the prioritisation of treatment targets in “third wave” interventions aimed at promoting acceptance of aversive internal experiences and decreased indiscriminate avoidant behaviours.

## Figures and Tables

**Table 1:** Binary logistic regression exploring whether versatility of methods, lifetime frequency, and recency of self-injury predict self-injury group (NSSI vs. NSSI + SB).

Characteristic	95% CI for OR			
	OR	Lower	Upper	*p*
Versatility ^a^	0.998	0.990	1.007	0.696
Lifetime frequency	1.000	0.997	1.002	0.767
Recency	1.000	1.000	1.000	0.838

R^2^ = 0.026 (Cox & Snell), 0.039 (Nagelkerke). Model χ^2^ (3) = 3.862, *p* = 0.277; ^a^ number of methods. Participants who indicated that they had ever self-injured engaged in an average of 3.69 methods of self-injury; CI: confidence interval; OR: odds ratio.

**Table 2:** Descriptive statistics and Jonckheere–Terpstra trend tests comparing experiential avoidance between Control, NSSI and NSSI + SB groups.

Variable	Control (C)		NSSI		NSSI + SB		T_JT_	*Z*	*p*	Ordered Effect
	*Mdn*	*IQR*	*Mdn*	*IQR*	*Mdn*	*IQR*				
Experiential avoidance (total) ^a,b^	119.00	51.00	129.00	47.75	128.50	49.00	13,655.00	1.90	0.058	-
Behavioural avoidance ^a,c^	36.00	13.50	38.00	11.00	39.00	15.50	15,512.00	1.89	0.058	-
Distress aversion ^a,d^	44.00	13.50	47.00	15.00	43.00	14.25	14,902.50	1.42	0.157	-
Procrastination ^a,c^	26.00	10.00	26.00	9.00	25.50	10.50	13,716.50	−0.38	0.707	-
Distraction/suppression ^a,e^	28.00	8.00	27.00	9.00	26.50	15.00	13,205.00	−0.85	0.397	-
Repression/denial ^a,f^	30.00	13.50	33.00	14.00	38.50	15.00	15,812.00	2.93	0.003 **	C < NSSI < NSSI + SB
Distress endurance ^a,g^	46.00	8.00	46.00	11.75	43.00	15.25	12,254.00	−1.814	0.070	-

^a^ As measured by the MEAQ; ** denotes significant at <0.01; ^b^ *n* = 291 ^c^ *n* = 310; ^d^ *n* = 308; ^e^* n* = 309; **^f^** *n* = 305; ^g^ *n* = 307; IQR: interquartile range, *Mdn*: median.
